# Priming maize resistance by its neighbors: activating 1,4-benzoxazine-3-ones synthesis and defense gene expression to alleviate leaf disease

**DOI:** 10.3389/fpls.2015.00830

**Published:** 2015-10-12

**Authors:** Xupo Ding, Min Yang, Huichuan Huang, Youcong Chuan, Xiahong He, Chengyun Li, Youyong Zhu, Shusheng Zhu

**Affiliations:** Key Laboratory of Agro-Biodiversity and Pest Management of Education Ministry of China, Yunnan Agricultural UniversityKunming, China

**Keywords:** intercropping, DIMBOA, resistance induction, defense genes, antimicrobial activity

## Abstract

Plant disease can be effectively suppressed in intercropping systems. Our previous study demonstrated that neighboring maize plants can restrict the spread of soil-borne pathogens of pepper plants by secreting defense compounds into the soil. However, whether maize plant can receive benefits from its neighboring pepper plants in an intercropping system is little attention. We examined the effects of maize roots treated with elicitors from the pepper pathogen *Phytophthora capsici* and pepper root exudates on the synthesis of 1,4-benzoxazine-3-ones (BXs), the expression of defense-related genes in maize, and their ability to alleviate the severity of southern corn leaf blight (SCLB) caused by *Bipolaris maydis*. We found that SCLB was significantly reduced after the above treatments. The contents of 1,4-benzoxazine-3-ones (BXs: DIBOA, DIMBOA, and MBOA) and the expression levels of BX synthesis and defense genes in maize roots and shoots were up-regulated. DIMBOA and MBOA effectively inhibited the mycelium growth of *Bipolaris maydis* at physiological concentrations in maize shoots. Further studies suggested that the defense related pathways or genes in maize roots and shoots were activated by elicitors from the *P. capsici* or pepper root exudates. In conclusion, maize increased the levels of BXs and defense gene expression both in roots and shoots after being triggered by root exudates and pathogen from neighboring pepper plants, eventually enhancing its resistance.

## Introduction

The capability of some plants to affect neighboring plants has been well documented (Broz et al., 2010; Ratnadass et al., 2012). In particular, certain plant diseases can be suppressed in biodiverse ecosystems (Zhu et al., 2000; Fan et al., 2010). This theory has been widely applied in traditional farming practices, such as intercropping, which has been practiced for 3000 years (de Albuquerque et al., 2010). In compatible intercropping systems, the crop productivity is increased (Li et al., 2009) and the development of wind-dispersed and soil-borne diseases and nematodes is suppressed (Wolfe, 1985; Zhu et al., 2000; Li et al., 2009; Newton and Guy, 2009; Dong et al., 2014; Gao et al., 2014; Yang et al., 2014). The mechanisms by which intercropping suppresses crop diseases are complicated; indeed, several mechanisms, such as inoculum dilution, spore dispersal interference, micro-environmental modification, and induced resistance, may be involved (Chin and Wolfe, 1984; Wolfe, 1985; Calonnec et al., 1996; Zhu et al., 2005; Boudreau, 2013). Recent studies have suggested that chemical defenses play an important role in suppressing disease in intercropping systems (Weston and Mathesius, 2013). For example, the crop roots secrete secondary metabolites, which can help the surrounding crops suppress soil-borne pathogen infection (Dixon, 2001; Gómez-Rodrıguez et al., 2003; Park et al., 2004; Bais et al., 2006; Basu et al., 2006; Ren et al., 2008; Bednarek and Osbourn, 2009; Frébortová, 2010). One crop release volatile organic compounds (VOCs) to help other crop to suppress wind dispersed pathogens (Kishimoto et al., 2005; Chehab et al., 2008; Yi et al., 2009; Du Fall and Solomon, 2011; Naznin et al., 2014).

Maize is the most popular crop used in intercropping systems. When maize was intercropped with legume, solanaceous, or cruciferous crops, the diseases of both intercropped crops were all significantly suppressed, as observed in intercropping with wheat and broad bean, maize and broad bean, maize and potato, maize and pepper, maize and tobacco, and maize and sugarcane (Li et al., 2007, 2009; Shukla et al., 2010). In maize and pepper intercropping system, maize plants can restrict the spread of soil-borne pathogens of pepper plants by secreting defense compounds into the soil. However, whether pepper plant can help its neighboring maize plants to suppress disease is little attention.

1,4-Benzoxazine-3-ones (BXs) are the most important secondary metabolites for defense in maize (Niemeyer, 2009). 2,4-Dihydroxy-7-methoxy-2H-1,4-benzoxazin-3(4H)-one (DIMBOA), which is derived from 2,4-dihydroxy-1,4-benzoxazin-3-one (DIBOA), is the major maize BX against herbivorous insects and pathogens (Friebe et al., 1998; Erb et al., 2009; Niemeyer, 2009; Ahmad et al., 2011). DIMBOA can be degraded into 6-methoxybenzoxazolin-3-one (MBOA), which also exhibits antimicrobial activity (Etzerodt et al., 2006; Maag et al., 2014). Previous studies have demonstrated that DIMBOA synthesis in maize can be regulated by biotic and abiotic elicitors (Niemeyer, 2009). The hormone-dependent defense pathways are also activated when DIMBOA in maize is induced by elicitors, such as NaCl or insects (Erb et al., 2009). Our previous study demonstrated that zoospores of the pepper pathogen *P. capsici* can be absorbed into the maize rhizosphere and ruptured by maize root exudates in maize and pepper intercropping system (Yang et al., 2014). It has been reported that the cell or the cell wall contents of *Phytophthora* can elicit plant defenses (Mozzetti et al., 1995; Gaulin et al., 2002; Xu et al., 2007). In addition, the root exudate of the surrounding plants contains ions, enzymes, mucilage, the primary and secondary metabolites may also have inductive effects on the surrounding plants (Bertin et al., 2003). Thus, root exudates and pathogens from the pepper plant may be potential elicitors for maize in an intercropping system of maize and pepper. However, whether the potential elicitors from pepper root exudates and the surrounding pathogens can induce the synthesis of BXs in maize tissues and improve their ability to defend themselves against leaf diseases remains to be elucidated.

Here, we assessed the profiles of BXs and defense-related genes in maize roots and shoots after treatment with intercropping elicitors from pepper root exudates and the pepper *Phytophthora* blight pathogen *P. capsici*. Our experiments suggested that the accumulation of BXs and the expression levels of defense genes in maize roots and shoots were induced when maize roots triggered by the elicitors form the pathogen and root exudates from the neighboring pepper plants, which could then enhance the resistance of maize against the above-ground leaf disease *B. maydis*.

## Materials and methods

### Plants and pathogens

Inbred B73 maize seeds were surface sterilized with 1% sodium hypochlorite for 5 min followed by four rinses in sterile distilled water. The seeds were then plated on wet cheesecloth, after which they were incubated in a growth chamber at 28°C for 7 days. The maize seedlings were transplanted into 500 mL sterile conical flasks with 200 mL 0.5% Hoagland fluid medium (only the maize plant roots were exposed to the medium) and incubated in a climate box at 28°C for 3 days with a 16 h light/8 h dark photoperiod (Janda et al., 1999). Each flask contained five maize seedlings at the three leaf stage and the medium was replaced in every day. The pepper seeds were grown in plastic pots containing an autoclaved sand-soil mixture for 30 days in a controlled climate chamber (28°C, with a 16 h light/8 h dark photoperiod) after surface sterilization with 1% sodium hypochlorite for 5 min.

The *P. capsici* (hereafter referred to as *PC*) was growth on carrot agar at 25 ± 1°C in petri plates for 7 days in darkness and incubated under fluorescent light at 28 ± 1°C for 48 h to induce sporulation. Zoospore release into 5 mL sterile water was induced by chilling harvested sporangia at 4°C for 40 min. The zoospore suspension was then diluted to 1 × 10^5^ spores·mL^−1^ with sterile water after the zoospores were counted with a cytometer under an optical microscope. *Bipolaris maydis* was grown on potato dextrose agar on petri plates for 7 days at 25 ± 1°C without light.

### Intercropping elicitor preparation, treatment, and sampling

The elicitors from *P. capsici* and pepper root exudate were prepared by the following methods: (1) the *PC* spore suspension (SP) was obtained by the methods described above and adjusted to 1 × 10^5^ spores·mL^−1^; (2) the *PC* spore lysis solution (SL) was obtained from the *PC* spore suspension (1 × 10^5^ spores·mL^−1^) after ultrasonic treatment in dark conditions for 2 h; (3) the *PC* spore culture suspension (CS) included the supernatants of the *PC* spore suspension (1 × 10^5^ spores·mL^−1^) centrifuged at 12,000 g after shaking in sterile water with 120 rpm for 2 h in dark conditions; (4) the healthy pepper root exudate (HRE) was collected from the exudate of 30 pepper plants growing in 200 mL sterile water for 8 h in light conditions, and the collected exudate was filtered with a 0.22 μm filter membrane (Millipore Express® PES membrane, Ireland); and (5) the nosogenic pepper exudate (NRE) was collected from the exudate of 30 pepper plants after pepper plant root infection by PC. The nosogenic pepper was obtained from pepper roots infected by PC zoospores (1 × 10^5^ spores·mL^−1^) for 5 days, after which these nosogenic plants were incubated in 200 mL sterile water for 8 h with light conditions. The NRE was filtered with a 0.22 μm filter membrane. Maize plants cultured as above were respectively transplanted into 500 mL sterile conical flasks with 200 mL above elicitor and incubated in a climate box at 28°C with a 16 h light/8 h dark photoperiod. The maize plants treated with sterile water was considered as the mock treatment. The elicitor medium was replaced per day. Three types of roots (crown-, primary-, and secondary roots) and shoots of maize plant were respectively collected at 0, 12, 24, 48, and 72 h after treatment with the above five elicitors. Each time point consisted of three replicates, and each replicate consisted of five seedlings. The roots and shoots were collected from each replicate, frozen in liquid nitrogen and stored at –80°C for the HPLC and gene expression analyses.

### Induced systemic resistance against *B. maydis*

Spores from the 7-day-old *B. maydis* culture were collected in sterile water (containing 0.1% Tween 20), and the spore concentration was adjusted to 5 × 10^4^ spores·mL^−1^. 10-day-old maize plant roots were induced with the above five elicitors or mock treated for 6 h. Then, 5 mL of spore suspension was sprayed onto the corresponding leaves of five maize plants with a 10 mL sprayer for each treatment. After inoculation, the plants were transplanted to the climatic box with a photoperiod of 16 h light/8 h dark and a relative humidity of 90% at 25°C. Five days after infection, disease lesions infected by *B. maydis* were quantified as described previously (Goodwin and Hsiang, 2010) by calculating the percentages of the lesion areas that accounted for the total leaf area. Each treatment included three replicates, and every replicate included five plants.

### Extraction and analysis of 1,4-benzoxazin-3-ones

To determine the concentrations of the three BXs derivatives DIBOA, DIMBOA, and MBOA in the shoots and different types of roots after treatment with the above five elicitors, a concise method was adopted as follows (Friebe et al., 1998). A total of 3–6 g of collected root or shoot samples was pulverized with liquid nitrogen, and 10 mL ethyl acetate was added directly. The homogenate was filtered and rotary evaporated at 37°C after homogenization. The residues were brought to a constant volume with 500 μL methyl alcohol (99%) and stored at –20°C for high-performance liquid chromatography (HPLC) analyses. To simultaneously and rapidly measure the three 1,4-benzoxazin-3-one derivatives in the roots and leaves, we developed a method based on the Agilent 1260 Infinity HPLC system equipped with a Kinetex C18 100 A column (100 × 4.6 mm, 2.6 μm) by monitoring the absorbance of elution at 254 nm. Solvent A is 0.1% phosphoric acid (Mallinckrodt Chemicals, LC/MS grade) in water (HPLC grade), and Solvent B is 99% methanol (OmniSolv, HPLC grade). The solvent gradient parameters were as follows: the gradient consisted of 0–10 min in 80–30% solution A, 10–10.5 min in 30% solution A, and 10.5–15.5 min in isocratic conditions of 100% in solution B. Five microlitres of the extracted sample was injected with a flow rate of 0.5 mL·min^−1^ at 30°C. With this method, the concentrations of DIBOA, BIMBOA, and MOBA from the crown, primary, and secondary roots and the leaves were quantified by standard curves, which showed linear relationships between the peak area and concentration. Each treatment included three replicates.

### Gene expression analysis in the maize roots and shoots

Total RNA was isolated from frozen root and shoot tissues using TRIzol Reagent (Qiangen, China) and quantified with a spectrophotometer (U-2910, HITCH, Japan). Four micrograms of total RNA was reverse transcribed by Easy-Script Reverse Transcriptase [M-MLV, RNase H^−^] and Oligo dT(18) primer (Transgen, China) at 42°C for 40 min and 85°C for 5 min according to the manufacturer's instructions. The reaction product cDNA was diluted into 50 ng·μL^−1^, and 1 μL was used for each semi-quantitative RT-PCR (semi-qPCR) reaction. The semi-qPCR reaction mix (50 μL) contained 1 μL of each gene-specific primer (10 μmol·μL^−1^), 8 μL of dNTPs, 5 μL of 10 × PCR buffer, 0.5 μL of Ex-Taq DNA polymerase (1 U·μL^−1^, Transgen, China) and 33.5 μL sterile water. The semi-qPCR included cDNA degenerated in 95°C for 5 min, with 25 cycles of 95°C for 30 s, 58°C for 30 s, and 72°C for 40 s, with extension at 72°C for 5 min in the ABI Veriti 96 system (Applied Biosystems, CA). The relative target gene expression levels were calculated by comparison with the maize housekeeping gene *GAPC*. The gene-specific primers used for all RT-PCR assays are listed in a Supplementary File (Table S1).

### Sensitivity testing of *B. maydis* to DIMBOA and MBOA

The sensitivities of *B. maydis* to DIMBOA and MBOA were determined according to a previous method (Mei et al., 2014). Briefly, a fresh plug (5 mm in diameter) was taken from the growing edge of a colony of *B. maydis* and cultured on potato dextrose agar (PDA) medium at 25°C for 4 days. These fungi were transferred onto PDA agar medium supplemented with DIMBOA (0, 1, 10, 20, 50, and 100 μg·mL^−1^) or MBOA (0, 2, 4, 40, 80, and 160 μg·mL^−1^), respectively. The concentrations of DIMBOA and MBOA were physiological concentrations of DIMBOA and MBOA for the maize shoots. The final amount of solvent did not exceed 1% (vol/vol) in the treated and control samples. Each treatment was replicated three times. After incubation at 25°C without light for 7 days for *B. maydis*, two measurements was taken for each colony at perpendicular angles, and the average of the two measurements was used for the inhibition ratio analysis. The diameter of the mycelium plug (0.5 cm) was subtracted from the colony diameter.

### Statistical analyses

All data obtained from the disease, antimicrobial activity and HPLC, were analyzed with Turkey *Post-hoc* ANOVA with SPSS 18.0 (*P* < 0.05). Then, Multibase 2014 was used for principal comparisons analyses (PLS-DA). The relative expression level of genes from semi-qPCR was analyzed with Hemi 2.0 after fold treatment with Quantity One from Bio-Rad and the hierarchical cluster was analyzed using Nearest neighbor with SPSS 18.0 (Deng et al., 2014).

## Results

### Induced systemic resistance against *B. maydis*

**Figure 1:**
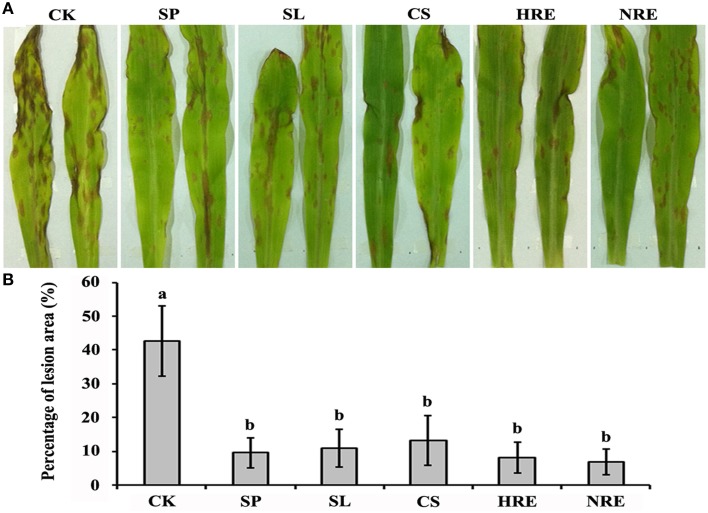
**Induced systemic resistance evaluation after maize roots induced by five elicitors. (A)** Symptoms on maize leaves infected with *B. maydis* after the roots were induced by five elicitors or mock treated; **(B)** The percentage of lesion area on the maize leaves (*n* = 5 plants per treatment, from three independent experiments). CK, mock treated; SP, spore suspension; SL, spore lysis suspension; CS, culture suspension; HRE, healthy root exudate; NRE, nosogenic root exudate. Treatments were statistically different (*p* < 0.05; Turkey *Post-hoc* ANOVA).

To investigate the induced systemic resistance against *B. maydis* in maize leaves, the lesions on leaves infected by *B. maydis* were quantified after the maize roots were induced by the spore suspension (SP), spore lysis suspension (SL), culture suspension (CS), healthy pepper root exudates (HRE), and nosogenic pepper root exudates (NRE). The lesions colonized by *B. maydis* were significantly reduced in all induced plants compared with the mock-induced plants (*p* < 0.05) (Figure 1). However, there were no significant differences among the five treatment groups (Figure 1).

### The accumulation of BXs in maize roots and shoots induced by five elicitors

To inspect the effects of the elicitors from neighboring pepper plants on the accumulation of defensive metabolites in maize, we profiled three BXs (DIBOA, DIMBOA, and MBOA) in crown-, primary-, secondary-, and shoots at 0, 12, 24, 48, and 72 h using HPLC analysis. Because the mock treatments at different time point did not show significant difference (Figure S1), we only analyzed the contents difference of BXs in shoots and roots at 0, 12, 24, 48, and 72 h after elicitor treatment.

**Figure 2:**
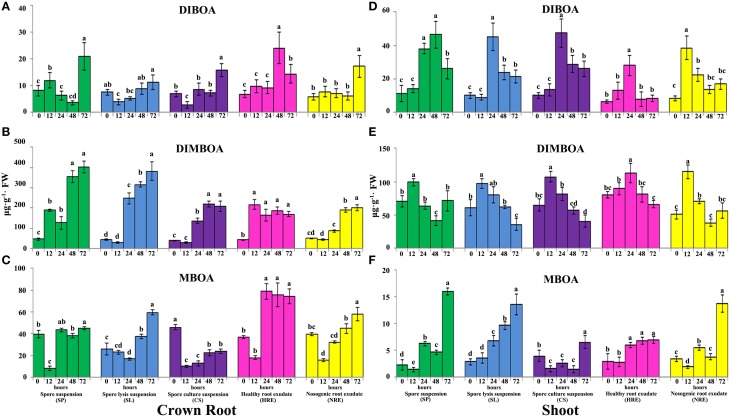
**Accumulation of DIBOA, DIMBOA, and MBOA in maize crown roots and shoots after treated with five elicitors**. Maize crown roots and shoots were collected at 0, 12, 24, 48, and 72 h post-elicitor induction. The content of DIBOA, DIMBOA, and MBOA in crown root **(A–C)** and shoot **(D–F)** was analyzed by HPLC (*n* = 3 replicates, with each replicate consisting of five plants). Treatments were statistically different (*p* < 0.05; Turkey *Post-hoc* ANOVA).

The accumulation of DIBOA in the roots and shoots was induced when the maize roots were treated with all elicitors (Figure 2, Figure S2). After treatment with SP, the content of DIBOA in the crown roots was significantly induced at 12 h, then significantly suppressed at 24 and 48 h. However, the content of DIBOA in the crown roots was significantly increased when checked at 72 h (Figure 2A). After treatment with SL and CS, the content of DIBOA in the crown roots was significantly decreased at 12 h, then increased with the extension of the treatment time (Figure 2A). The accumulation of DIBOA in primary and secondary roots showed similar trend with crown roots after treated with SP, SL, and CS (Figure S2A). Furthermore, the contents of DIBOA in the primary and secondary roots were significantly lower than in paired crown roots (*p* < 0.05). The content of DIBOA in the shoots was significantly increased after the maize roots were treated with SP, SL, and CS at 24 or 48 h but then decreased (Figure 2D). After treatment with HRE and NRE, the content of the DIBOA in the roots and shoots was also significantly induced. The response of DIBOA concentration in the roots to HRE was earlier than the response to NRE (Figure 2D). However, the response of the accumulation of DIBOA in the shoots to HRE occurred later than the response to NRE (Figure 2D). PCA analysis indicated that the synthesis profiles of DIBOA in the crown-, primary-, secondary roots and shoots after treatment with HRE were separated from the other four elicitors (Figures S3A–D). The synthesis profile of DIBOA in the crown- and primary roots after treatment with NRE was not significantly separated from SP, SL, and CS on the PC1 direction (Figures S3A,B). In addition, the synthesis profiles of DIBOA in the crown-, primary-, secondary roots and shoots overlapped after treatment with SP, SL, and CS (Figures S3A–D).

The content of DIMBOA in the crown roots was significantly induced after treatment with SP, SL, and CS from 12 to 72 h (Figure 2B). However, the content of DIMBOA in the shoots was significantly increased at 12 h and then decreased from 24 to 72 h (Figures 2E). The accumulation of DIMBOA in primary and secondary roots showed similar trends with crown roots after treatment with SP, SL, and CS (Figures S2B,E). After treatment with HRE, the DIMBOA content of the crown roots was significantly induced, and this content was consistent from 12 to 72 h (Figure 2B). The accumulation of DIMBOA in the primary-, secondary roots, and shoots showed a similar trend, in which the content was significantly increased at 12 and 24 h and then significantly decreased (Figure 2B, Figures S2B,E). After treatment with NRE, the DIMBOA content in the crown-, primary-, and secondary roots was not found to be significantly increased at 12 or 24 h (Figure 2B, Figures S2B,E). However, the DIMBOA content of the shoots was significantly increased at 12 h and then decreased from 24 to 72 h (Figure 2E). PCA analysis demonstrated that the synthesis profiles of DIMBOA in the crown, primary, and secondary roots after treatment with HRE are also separated from other elicitors (Figures S3E–G). However, the synthesis profile of DIMBOA in the crown roots treated by NRE was not significantly separated from the SL and CS in terms of the PC1 direction. In addition, the synthesis profile of DIMBOA in the shoots after treatment with HRE was separated from that of the other four elicitors. The DIMBOA synthesis profiles of the shoots after treatment with NRE overlapped with the treatment of SP, and the treatment of SL also overlapped with the treatment of CS (Figure S3H).

Similarly, the accumulation of MBOA in the roots and shoots was modified by the five elicitors. After treatment with SP, the MBOA content in the roots and shoots was significantly decreased at 12 h (Figures 2C,F, Figures S2C,F), except in the secondary roots, its content then significantly increased at 24 h, with the highest content in roots and shoots at 72 h. After being treated with SL, the MBOA content in the crown and secondary roots decreased from 12 to 24 h and increased from 48 to 72 h (Figure 2C, Figure S2F). The MBOA content of shoots was significantly increased from 24 to 72 h (Figure 2F). After treatment with CS, the MBOA content in the roots and shoots was significantly increased at 72 h, except in the crown roots, in which the MBOA content was significantly below that of the control (Figure 2C). After treatment with HRE and NRE, the content of MBOA in the crown roots was significantly decreased at 12 h and then increased from 24 to 72 h (Figure 2C). The content of MBOA in the primary and secondary roots and shoots increased from 12 to 72 h (Figures S2C,F, Figure 2F). PCA analysis revealed that the synthesis profiles of MBOA in the roots and shoots after the treatment with the root exudates were separated from those of the other elicitors in the PC1 direction (Figures S3I–L). The synthesis profiles of MBOA in the crown, primary and secondary roots after treatment with HRE and NRE were overlapped but separated from those of the other elicitors in the PC2 direction (Figures S3I–K). The synthesis profiles of MBOA in the crown roots and shoots after treatment with SP and NRE were not separated in the PC1 direction. The synthesis profiles of MBOA in the crown- and primary roots after treatment with SP and CS were not separated in the PC2 direction (Figures S3I,J).

### DIMBOA and MBOA exhibit antimicrobial activity against and *B. maydis*

**Figure 3:**
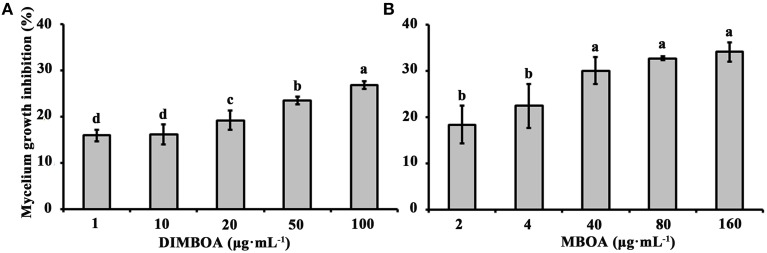
**Antimicrobial activities of DIMBOA and MBOA against the mycelium growth of ***B. maydis*****. Average mycelium growth inhibition (±SE) of *B. maydis* observed on media supplemented with different concentrations of DIMBOA **(A)** or MBOA **(B)**. Different letters indicate significant differences between the mycelium growth inhibition at different concentrations of DIMBOA or MBOA. Treatments were statistically different (*p* < 0.05; Turkey *Post-hoc* ANOVA).

To determine whether physiological concentrations of DIMBOA and MBOA in maize shoots have antimicrobial activity, the mycelium growth of *B. maydis* was tested on artificial media mixed with different concentrations of DIMBOA (1–100 μg·mL^−1^) and MBOA (2–160 μg·mL^−1^), respectively. This experiment showed that DIMBOA and MBOA displayed antimicrobial activity and could inhibit the mycelium growth of maize pathogen *B. maydis* (Figure 3). The mycelium growth of *B. maydis* was significantly inhibited when DIMBOA reached a concentration of 20 μg·mL^−1^ (*p* < 0.05) (Figure 3A). The mycelium growth of *B. maydis* was significantly suppressed by MBOA at a concentration of 40 μg·mL^−1^ (*p* < 0.05) (Figure 3B).

### The expression profiles of BX synthesis and defense-related genes in maize crown roots and shoots after treatment with the five elicitors

To determine whether the elicitors from neighboring pepper plants induced the expression of BX synthesis and defense-related genes in maize, we analyzed 21 stress- or hormone-induced genes in crown roots and shoots after roots were treated with five elicitors from 12 to 72 h and checked by semi-quantitative RT-PCR (Figure S4).

**Figure 4:**
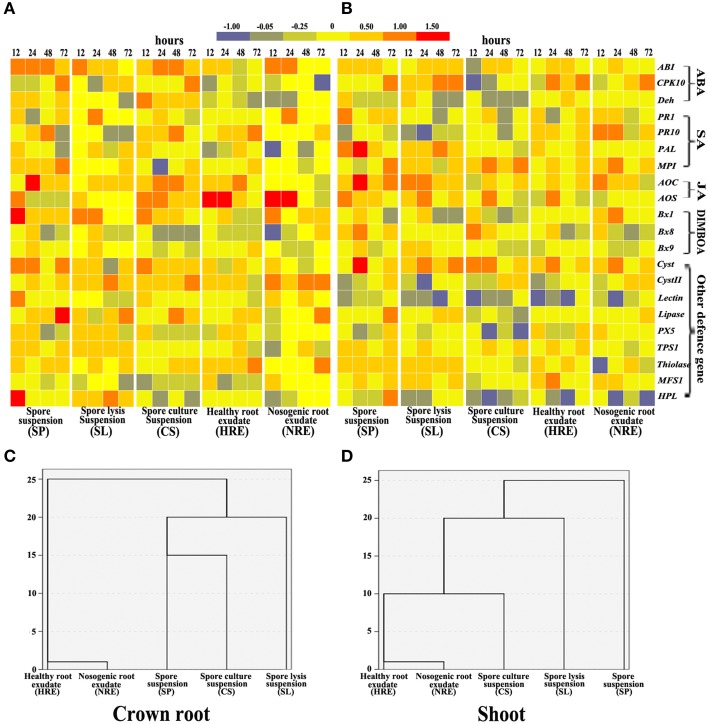
**Gene expression profiles in maize crown roots and shoots from 12 to 72 h of maize roots treated with five elicitors**. Maize was collected at 0, 12, 24, 48, and 72 h post-elicitor induction. Gene expression in maize crown roots **(A)** and shoots **(B)** were indicated in terms of the fold induction compared to the mock-treated plant, and the bars show their fold changes. The 21 stress gene expression profiles in crown roots **(C)** and shoots **(D)** were analyzed by hierarchical cluster analysis (*n* = 3 replicates, with each replicate consisting of five plants).

In crown roots, the expression of ABA, SA, and JA pathway related genes were activated by both root exudates and *PC*-related elicitors, whereas the ABA pathway was strongly activated by PC-related elicitors and the JA pathway was strongly activated by elicitors from root exudates (Figure 4A). In shoots, the expression of ABA, SA, and JA pathway related genes was also induced by all five elicitors with different degrees (Figure 4B). The SA pathway was strongly activated by root exudates, whereas JA pathway was strongly activated by *PC*-related elicitors (Figure 4B). The expression of BXs synthesis genes in crown roots and shoots were also induced by all five elicitors with different degrees (Figures 4A,B). Almost all other defense genes except *MFS1* in crown shoots were activated by all five elicitors, whereas only *Cyst, TPS1, Thiolase*, and *MFS1* were activated in shoots (Figures 4A,B).

The hierarchical cluster analysis data indicated that BX synthesis genes and defense-related genes were activated by five elicitors with different patterns. The profiles induced by SP, SL, and CS clearly clustered away from the profiles induced by HRE and NRE in the crown roots (Figure 4C) and shoots (Figure 4D). Although the expression of BX synthesis and defense-related genes in primary and secondary roots were also activated by both root exudates and PC-related elicitors (Figures S5A,B), the gene expression profiles induced by *PC*-related elicitors did not clearly clustered away from the profiles induced by root exudates related elicitors in primary root and secondary roots (Figures S5C,D).

## Discussion

Plant diseases and insects can be effectively controlled in complicated and diverse ecosystems (Knops et al., 1999; Zhu et al., 2000). Intercropping is the classical applied technology of biodiversity in agroecosystems (Li et al., 2009). Intercropping not only improves plant growth and productivity but also enhances plant resistance to various insects and pathogens (Altieri, 1999; Li et al., 2007; Boudreau, 2013; Gao et al., 2014). In our previous field studies, intercropping was confirmed to suppress maize and pepper disease through inoculum dilution, spore dispersal interference, micro-environmental modification, and allelopathy (Li et al., 2009; Yang et al., 2014). Studies have demonstrated that the below-ground part of the maize is infected by insects or colonized by plant growth-promoting rhizobacteria (PGPR), which trigger above-ground resistance against worms or leaf diseases (Erb et al., 2009, 2011; Song et al., 2011). In the present study, we found that the resistance of maize against the above-ground leaf disease *B. maydis* was enhanced when its roots were induced by non-host pathogen *P. capsici* or root exudates from pepper plants (Figure 1). Induced systemic resistance (ISR) in plants is recognized to be effective against a wide range of pathogen infections (Ryals et al., 1996; Van Loon et al., 1998; Naoumkina et al., 2007; Shoresh et al., 2010). The induced defense was sensitized by the plant response to biotic and abiotic elicitors (Maldonado-Bonilla et al., 2008). Studies have indicated that plant pathogens were suppressed when plant roots were inoculated with non-adapted bacteria, fungi, or viruses or treated with exogenous root exudates (Pozo et al., 2002; Weller et al., 2012). Even microbial cell wall-related substances can induce systemic acquired resistance in plants (Zhao et al., 2007; Sillero et al., 2012; Nawrocka and Małolepsza, 2013). The synthesis of defensive substances and the expression of defense-related genes are two important mechanisms involved in ISR (Maldonado-Bonilla et al., 2008). In the present study, we found that the BXs accumulation and defense-related genes expression in maize shoots and roots were enhanced when the maize roots were induced by elicitors.

BXs are the key defense substances in maize (Niemeyer, 2009; Ahmad et al., 2011). Studies have demonstrated that BXs have antifungal activity against phytopathogenic fungi and can be induced by a trigger, such as aphids, worms, pathogens, or PGPR (Ahmad et al., 2011; Neal et al., 2012; Robert et al., 2012; Betsiashvili et al., 2015). Here, we found that the accumulation of BXs in maize roots and shoots could be induced by the spore suspension, spore lysis suspension, and culture suspension from *P. capsici* and the root exudates from pepper plants. In particular, the accumulation of DIMBOA in the crown roots was found to be up-regulated by five- to ten-fold after induction by *PC* or pepper root exudates related elicitors for 72 h (Figure 2A). The concentration of DIBOA in the shoots was significantly increased to 10–40 or 50–100 μg·mL^−1^ and the concentration of MBOA reached 20 μg·mL^−1^ at 72 h (Figure 2B). Our antifungal activity test demonstrated that the mycelium growth of the maize pathogen *B. maydis* were significantly inhibited by DIMBOA and MBOA when their concentrations reached 20 and 40 μg·mL^−1^, respectively (Figure 3). These data indicated that the reduced lesions in shoots infected by *B. maydis* might be due to the building up of DIMBOA and MBOA in the shoots after being triggered by elicitors. However, the expression of other defense-related genes in shoots might also involve in the resistance of maize to *B. maydis*. It has been reported that other defense-related genes can be regulated in maize by biotic or exogenous chemical elicitors (Yang et al., 2012; Sommer et al., 2014). Here, we found that *Cyst, TPS1, Thiolase*, and *MFS1* were strongly activated in shoots after treatment with all five elicitors.

The induced profiles of BXs in maize treated with elicitors from nosogenic pepper root exudates were different from those treated with elicitors from healthy pepper root exudates but similar to those treated with *PC*-related treatments, especially on the DIBOA and DIMBOA. This finding may be due to differences in the substances secreted by the nosogenic and healthy pepper roots. Previous studies have demonstrated that defense substances in plants, such as momilactone, sakuranetin, camalexins, capsidiol, resveratrol, and piceids, can be induced by fungi, bacteria, or their cell walls (Ma, 2008; Jasiński et al., 2009; Mialoundama et al., 2009; Yang et al., 2010). Interestingly, the fusaric acid produced by *Fusarium* spp. also induced the accumulation of camalexin in Arabidopsis (Bouizgarne et al., 2006). In addition, certain compounds separated from plant root exudates, such as plant hormones and secondary metabolites, can induce the resistance of the plant against pathogen infection (Wen et al., 2007; De-la-Peña et al., 2008). It has also been reported that the elicitors from plants infected by insects or pathogens can effectively enhance the production of defense or signaling substances used to help the plant or a neighboring plant activate its defense-related pathways (Engelberth et al., 2004; Ton et al., 2007; Ahuja et al., 2012). These studies demonstrated that some special elicitors, which were contained in the *P. capsici* and pepper root exudates, could induce BX synthesis in roots and shoots. However, the special elicitors contained in the *P. capsici* and root exudates should be further identified.

The transcripts of *Bx1, Bx8*, and *Bx9*, which are the key genes for DIMBOA synthesis in maize, were up-regulated in roots and shoots after the maize roots were treated by elicitors, suggesting that the DIMBOA synthesis gene expression was consistent with the accumulation of BXs in roots and shoots. Furthermore, the BX synthesis gene was reported to be synchronously regulated with certain defense-related genes, such as the JA-related gene *AOS*, the SA-related *PR* genes, the ABA-related gene *ABI* and 9-cis-epoxycarotenoid dioxygenase (Farag et al., 2005; Alleman et al., 2006; Schnee et al., 2006; Erb et al., 2009; Frey et al., 2009). The latest studies have demonstrated that BX synthesis genes in maize, especially *Bx1, Bx8*, and *Bx9*, can be regulated by ABA, NaCl, insects or PGPR (Erb et al., 2009, 2011; Song et al., 2011; Planchamp et al., 2015). In this study, the expression pattern of *Bx1* was similar to *ABI*, which may imply that *Bx1* expression was synchronously regulated by ABA pathway. However, it should be further identified with mutant plant.

For maize, defense genes such as ABA-related genes (*ABI, CPK*10, and *Deh*), SA-related genes (*PR1, PR10, PAL*, and *MPI*), and JA-related genes (*AOC* and *AOS*) can be regulated by insects, pathogens or exogenous ABA, BTH, JA, or NaCl (Ton et al., 2007; Erb et al., 2009; Lanubile et al., 2012; Kumar et al., 2014). Furthermore, some defense-related genes (*Cys, Cys II, Lectin, PX5, TPS, Thiolase, MFS1*, and *HPL*) that do not belong to the major hormone-related signaling pathways can be regulated in maize by biotic or exogenous chemical elicitors (Yang et al., 2012; Sommer et al., 2014). Our wide spectrum of defense-related gene expression profiles demonstrated that all five elicitors can regulate defense gene expression with different degrees (Figure 4). Generally, the expression profiles induced by the *PC-*related elicitors were different from those induced by pepper root exudates in crown roots and shoots (Figures 4C,D). ABA pathway in roots and shoots can be induced by all elicitors, whereas JA pathway in shoots was strongly induced by PC-related elicitors and SA pathway in shoots was strongly by pepper root exudates (Figure 4B). In crown roots, the ABA pathway was strongly activated by PC-related elicitors whereas JA pathway was strongly activated by elicitors from root exudates (Figure 4A). Previous studies have indicated that the ABA pathway is essential in response to biotic stress and negatively regulates the SA pathway in plants. These studies also demonstrated the existence of a synergistic interaction between the SA and JA pathways and an interference with ABA and JA pathways (Robert-Seilaniantz et al., 2007; Lee and Luan, 2012; Montillet and Hirt, 2013). However, we did find significant synergistic or interference interaction between ABA, JA, and SA pathway in this study. In addition, the expression differences in the defense-related genes might be due to the different elicitors secreted by *P. capsici* and the pepper root exudates. In general, plant pathogens secrete many special enzymes, saccharides, toxins, and other organic substances (Xu and Du, 2006). Some components are even released from the cell wall, entocyte, or liptocoenosis when the pathogen is dead (Mattoo et al., 2007; Giraud et al., 2010). Plant roots secrete ions, inorganic acids, amino acids, volatile compounds, mucilage, and proteins (Bais et al., 2006; Uren, 2007). Many of these substances have been reported to induce plant defense-related gene expression in different hormone-related pathways (Okubara and Paulitz, 2005; Baldi et al., 2008; Huang et al., 2014). Therefore, the elicitors existing in *P. capsici* and the pepper root exudates may differ. Further experiments are planned to confirm this hypothesis.

Although the accumulation of BXs and the expression of defense-related genes were induced in three types of roots after treated with five elicitors, but the induced profiles in crown roots by five elicitors were different with their profiles in primary and secondary roots (Figure S5). This might be due to the different function of root types. The maize crown root, which contains higher concentration of carbohydrates, amino acids, and defensive substances, are more important for young maize plant development and defense in young plant than primary and secondary root (Erb, 2012; Robert et al., 2012). In this study, we found that the accumulation of BXs in crown roots was higher than in primary and secondary roots after treated with elicitors, which further demonstrated that crown root was the important root type for plant defensive.

In conclusion, maize increased the levels of BXs and defense gene expression both in roots and shoots after being triggered by elicitors from neighboring pepper plants, which might eventually enhance its resistance to the leaf pathogen *B. maydis*. These data showed that the elicitor-induced resistance from the neighboring plants was an important mechanism in the suppression of plant disease in the intercropping system.

## Author contributions

SZ and XD designed the research; XD, MY, and YC performed the research; XD and HH analyzed the data; SZ and XD wrote the paper; XH, CL, and YZ reviewed the paper.

### Conflict of interest statement

The authors declare that the research was conducted in the absence of any commercial or financial relationships that could be construed as a potential conflict of interest.

## Supplementary material

Figure S1 **Accumulation of BXs (DIBOA, DIMBOA, and MBOA) in maize shoot and roots after treated with sterile water as the mock treatment**. Maize shoots and roots were collected at 0, 12, 24, 48, and 72 h post-sterile water treated. The content of DIBOA, DIMBOA, and MBOA in maize shoots, crown roots, primary roots and secondary roots was analyzed by HPLC (*n* = 5 replicates, with each replicate consisting of five plants). Treatments were statistically different (*p* < 0.05; Turkey *Post-hoc* ANOVA). **(A–D)** DIBOA; **(E–H)** DIMBOA; **(I–L)** MBOA.

Figure S2 **Accumulation of DIBOA, DIMBOA, and MBOA in maize primary and secondary roots after treated with five elicitors**. Maize primary roots and secondary roots were collected at 0, 12, 24, 48, and 72 h post-elicitor induction. The content of DIBOA, DIMBOA, and MBOA in primary root **(A–C)** and secondary root **(D–F)** was analyzed by HPLC (*n* = 3 replicates, with each replicate consisting of five plants). Treatments were statistically different (*p* < 0.05; Turkey *Post-hoc* ANOVA).

Figure S3 **Principal component analysis of the DIBOA, DIMBOA, and MBOA content of maize roots and shoots**. DIBOA **(A–D)**, DIMBOA **(E–H)** and MBOA **(I–L)** in maize crown, primary, secondary root and shoots. Green, blue, purple, magenta, and yellow images denote treatment with SP, SL, CS, HRE, and NRE, respectively.

Figure S4 **The electrophoresis gels of 21 stress genes expression in maize roots and shoots from 0 to 72 h after maize roots treatment with five elicitors**. Maize was collected at 0, 12, 24, 48, and 72 h post-elicitor induction. **(A)** Shoots, **(B)** crown roots, **(C)** primary roots, **(D)** secondary roots.

Figure S5 **Gene expression profiles in maize primary and secondary roots from 12 to 72 h of maize roots treatment with five elicitors**. Maize was collected at 0, 12, 24, 48, and 72 h post-elicitor induction. Gene expression in maize primary roots **(A)** and secondary roots **(B)** were indicated in terms of the fold induction compared to the mock-treated plant, and the bars show their fold changes. The 21 stress gene expression profiles in primary roots **(C)** and secondary roots **(D)** were analyzed by hierarchical cluster analysis (*n* = 3 replicates, with each replicate consisting of five plants).

Table S1 **Gene names, putative function and corresponding primer sequences of genes used in this study for semi-qPCR**.
