# Supplementary figures and images for: Priming maize resistance by its neighbors: activating 1,4-benzoxazine-3-ones synthesis and defense gene expression to alleviate leaf disease
Source: Front Plant Sci. 2015 Oct 12;6:830. doi: 10.3389/fpls.2015.00830 (PMC4600908; doi:10.3389/fpls.2015.00830)

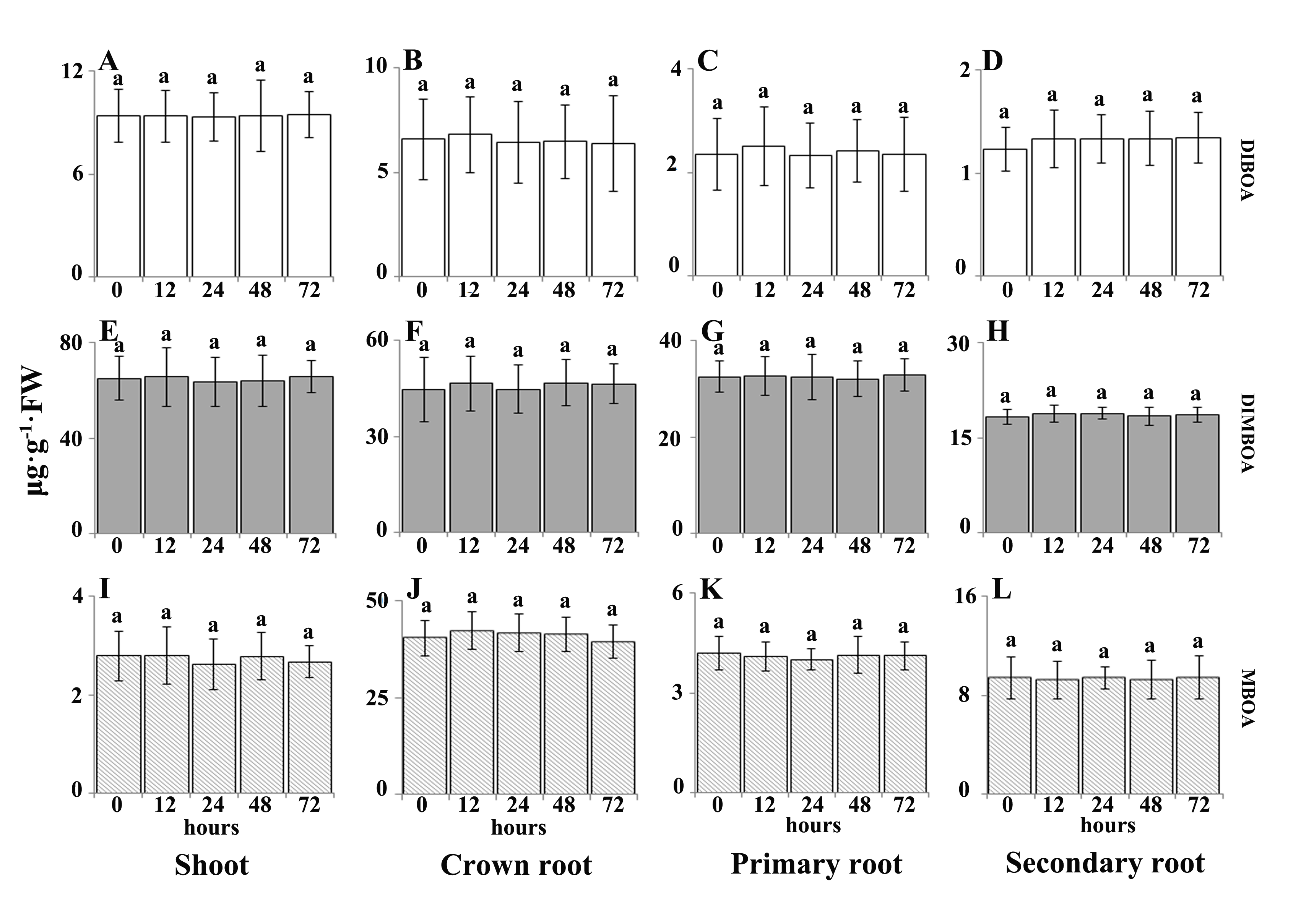

Supplement: Figure S1 — Accumulation of BXs (DIBOA, DIMBOA, and MBOA) in maize shoot and roots after treated with sterile water as the mock treatment. Maize shoots and roots were collected at 0, 12, 24, 48, and 72 h post-sterile water treated. The content of DIBOA, DIMBOA, and MBOA in maize shoots, crown roots, primary roots and secondary roots was analyzed by HPLC (n = 5 replicates, with each replicate consisting of five plants). Treatments were statistically different (p < 0.05; Turkey Post-hoc ANOVA). (A–D) DIBOA; (E–H) DIMBOA; (I–L) MBOA. [file Image1.TIF]

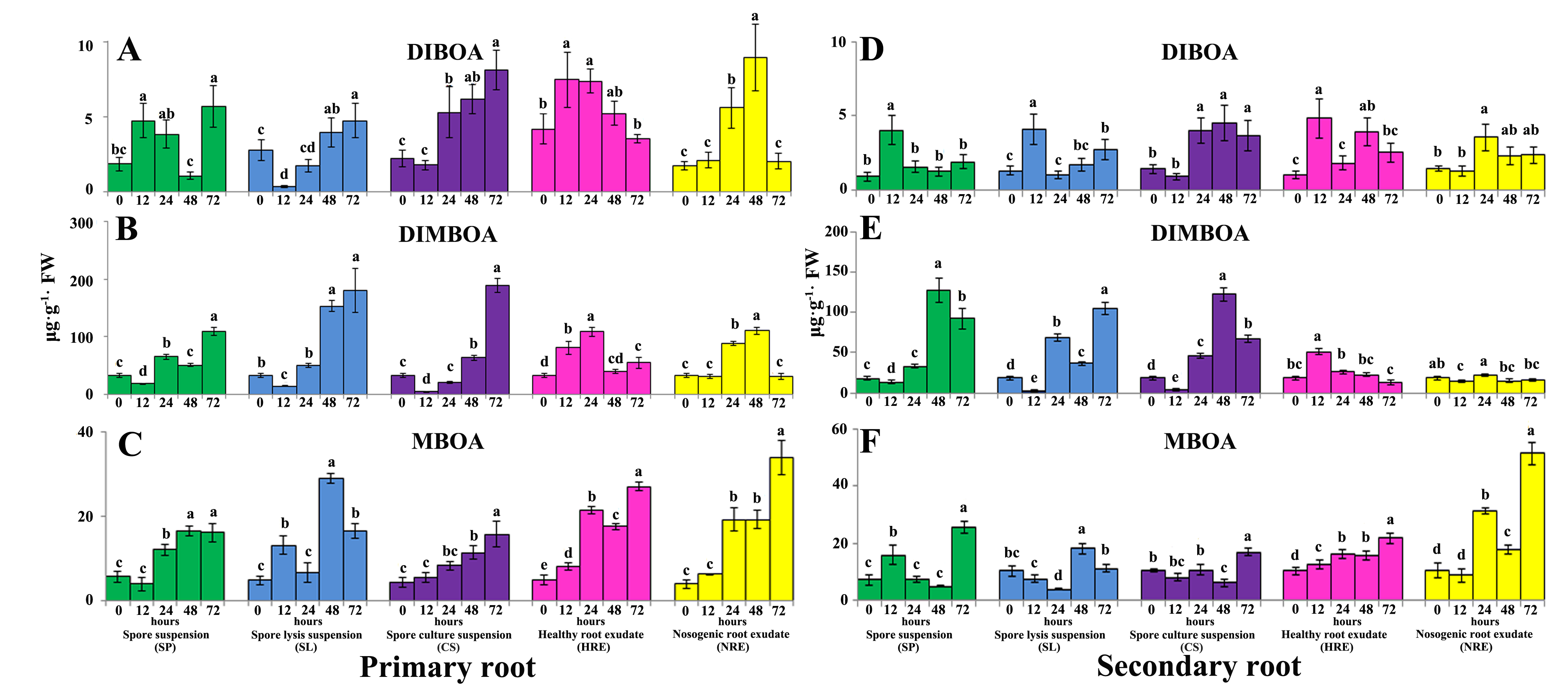

Supplement: Figure S2 — Accumulation of DIBOA, DIMBOA, and MBOA in maize primary and secondary roots after treated with five elicitors. Maize primary roots and secondary roots were collected at 0, 12, 24, 48, and 72 h post-elicitor induction. The content of DIBOA, DIMBOA, and MBOA in primary root (A–C) and secondary root (D–F) was analyzed by HPLC (n = 3 replicates, with each replicate consisting of five plants). Treatments were statistically different (p < 0.05; Turkey Post-hoc ANOVA). [file Image2.TIF]

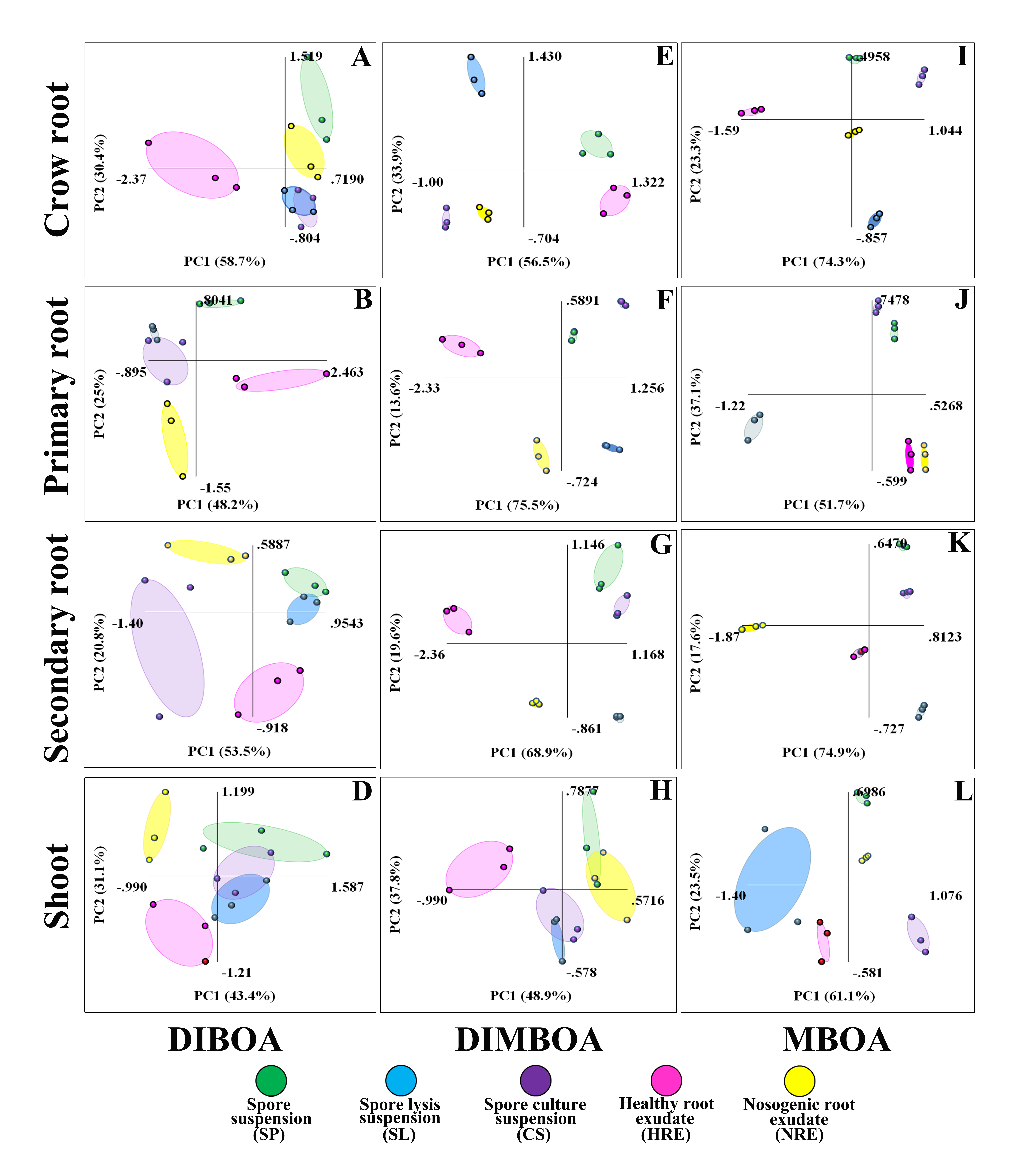

Supplement: Figure S3 — Principal component analysis of the DIBOA, DIMBOA, and MBOA content of maize roots and shoots. DIBOA (A–D), DIMBOA (E–H) and MBOA (I–L) in maize crown, primary, secondary root and shoots. Green, blue, purple, magenta, and yellow images denote treatment with SP, SL, CS, HRE, and NRE, respectively. [file Image3.TIF]

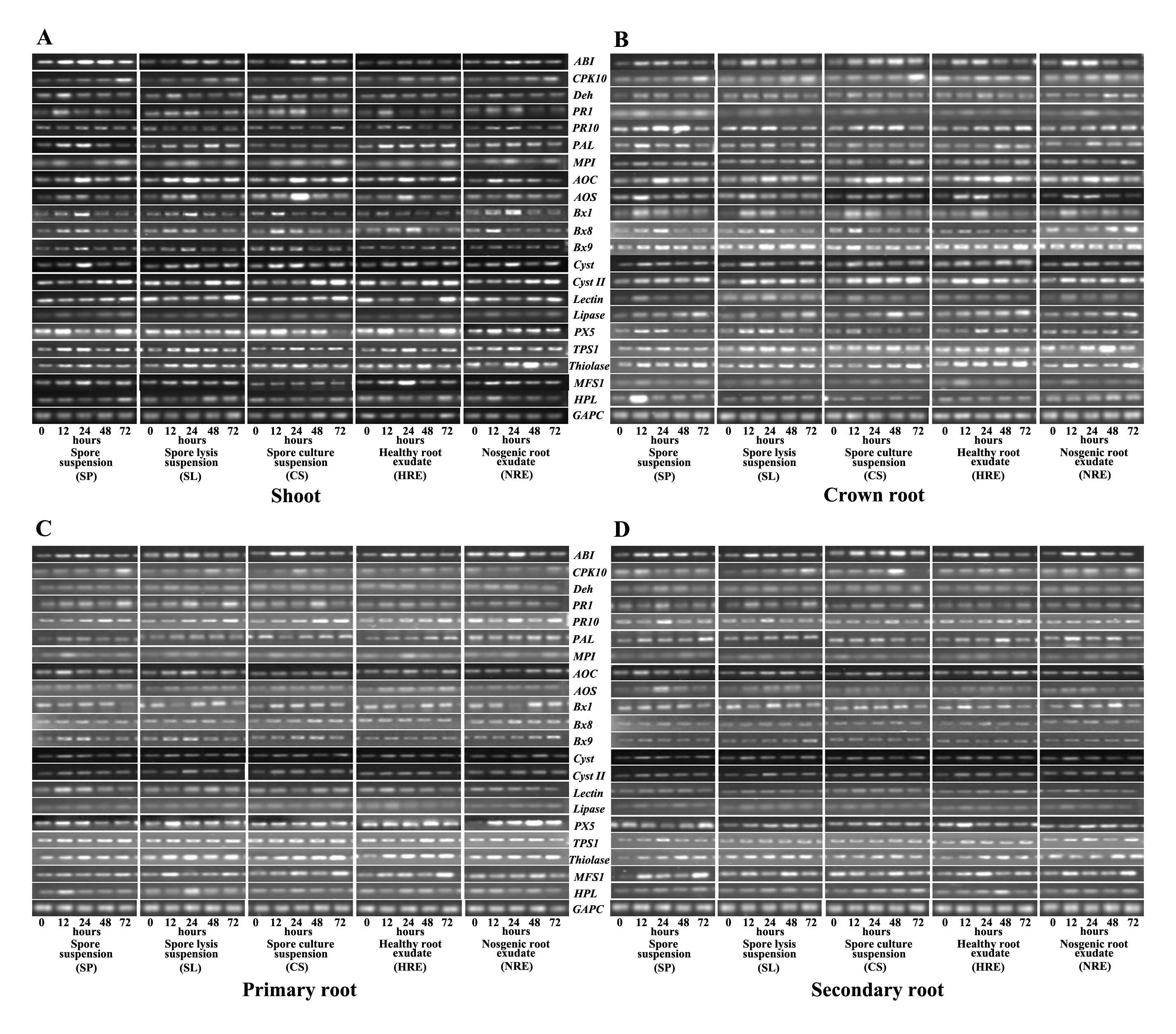

Supplement: Figure S4 — The electrophoresis gels of 21 stress genes expression in maize roots and shoots from 0 to 72 h after maize roots treatment with five elicitors. Maize was collected at 0, 12, 24, 48, and 72 h post-elicitor induction. (A) Shoots, (B) crown roots, (C) primary roots, (D) secondary roots. [file Image4.TIF]

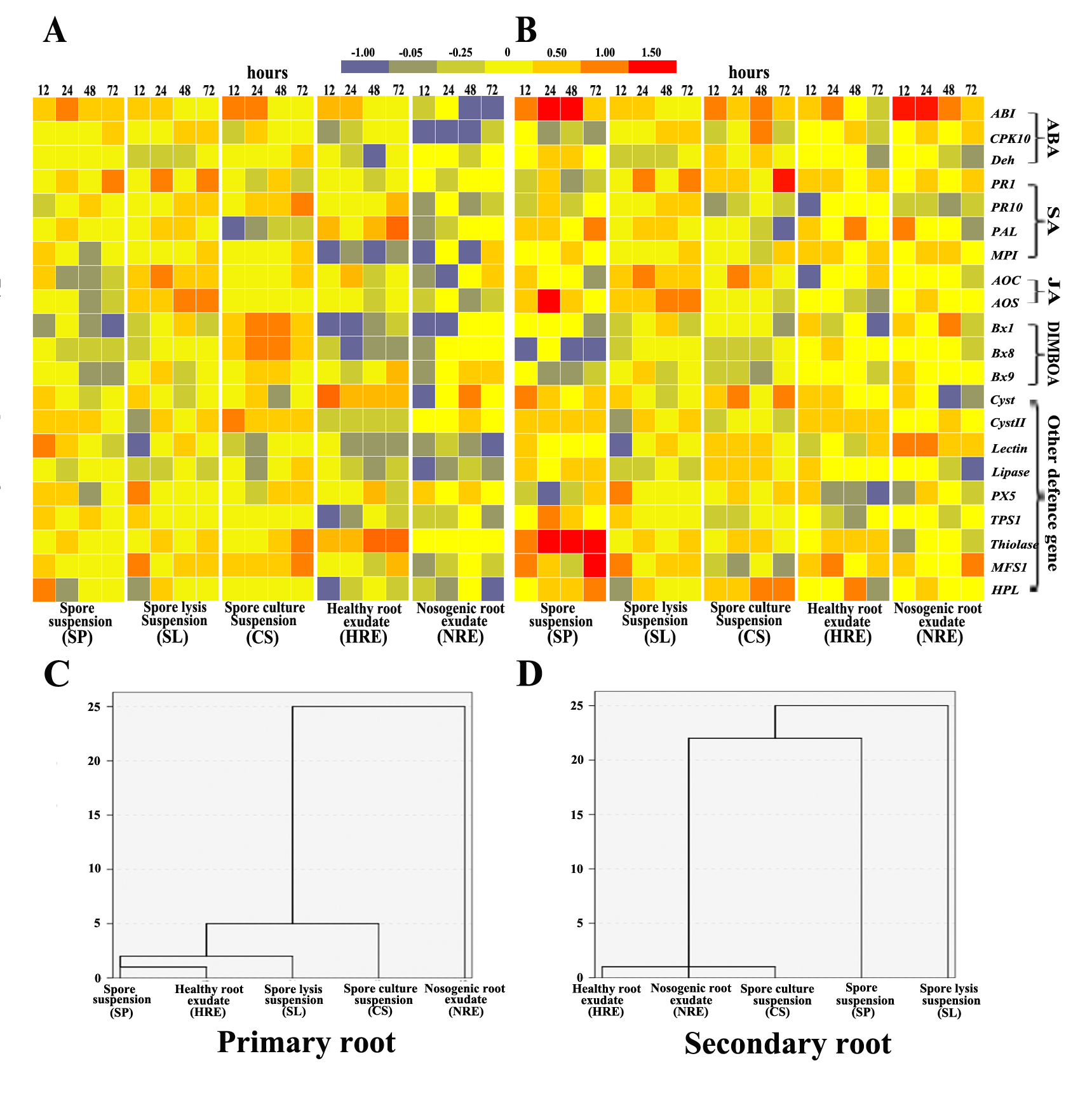

Supplement: Figure S5 — Gene expression profiles in maize primary and secondary roots from 12 to 72 h of maize roots treatment with five elicitors. Maize was collected at 0, 12, 24, 48, and 72 h post-elicitor induction. Gene expression in maize primary roots (A) and secondary roots (B) were indicated in terms of the fold induction compared to the mock-treated plant, and the bars show their fold changes. The 21 stress gene expression profiles in primary roots (C) and secondary roots (D) were analyzed by hierarchical cluster analysis (n = 3 replicates, with each replicate consisting of five plants). [file Image5.TIF]
