# Supplementary material for: Priming maize resistance by its neighbors: activating 1,4-benzoxazine-3-ones synthesis and defense gene expression to alleviate leaf disease
Source: Front Plant Sci. 2015 Oct 12;6:830. doi: 10.3389/fpls.2015.00830 (PMC4600908; doi:10.3389/fpls.2015.00830)
Supplement: Table S1 — Gene names, putative function and corresponding primer sequences of genes used in this study for semi-qPCR. [file Table1.DOCX]

**Table S1. Primer sequences of DIMBOA synthesis and defense-related genes in maize that**

**were used in this study for RT-qPCR and semi-qPCR analysis**

| **Gene name** | **Putative function** | **Left primer** | **Right primer** |
| --- | --- | --- | --- |
| *ABI* | Homology to glycin-rich protein | gcgagatcctcgactccaag | gggcttggttaacggtgatg |
| *AOC* | Allene oxide cyclase | ccccttcaccaacaaggtgt | accgagatgtggccgtagtc |
| *AOS* | Allene oxide synthase | acctgttcacgggcacctac | cgaggagcgaggagaagttg |
| *Bx1* | DIMBOA biosynthesis | cccgagcacgtaaagcagat | cttcatgcccctggcatact |
| *Bx8* | DIMBOA biosynthesis | gatacctgccggtgagagag | gggaacgtgtggaagatgag |
| *Bx9* | DIMBOA biosynthesis | gcaacatgaggtacgtgtgc | gcagcgatcttgaattcctt |
| *CPK10* | Calcium-dependent protein kinase | gaggtacgtgtgc | cggatgccgtagaacttcgt |
| *Cyst2* | Cystatin II proteinase inhibitor | tgccctgctcatactgcttg | gcgagttcctggaggtgaag |
| *Deh* | Dehydrin | accagtacggcaacccagtc | gccggtcttgtgctcctc |
| *GAPC* | Glyceraldehyde Glyceraldehyde phosphate dehydrogenase | gcatcaggaaccctgaggaa | catgggtgcatctttgcttg |
| *HPL* | Hydroperoxide lyase | acttcggcttcaccatcctg | gtagtagcccggccagatga |
| *Lectin* | Lectin | tcgtcgtccttggagagctt | catctgccaagtccccttct |
| *Lipase* | Lipase/esterase | ccaagagcctcatcatcgtg | cgtggtagtggtccgtgttg |
| *MFS* | Multiflux efflux synthase | cactgtgggctgtgagcagt | gcaggccgaaatgtcttgat |
| *PAL* | phenylalanine ammonia lyase | aagaaggtgaacgagctgga | gttgtcgttcacggagttga |
| *PR1* | Pathogenesis-related gene 1 | ctgggtgtccgagaagcagt | cgggttgtagctgcagatgat |
| *PR10* | Pathogenesis-related gene 10 | gtcatgccgttcagcttcat | tgttcttgcactcgacttg |
| *Px5* | Peroxidase | ggattgatcctgcgctgag | gactcgaagaggcccaggtt |
| *Cyst* | Cystatin-like proteinase inhibitor | agggcttgttcggttaggtg | tgcagaataaggagccatgc |
| *Thiolase* | Thiolase | ttcgcccaagtttcaaggag | gccgcatctgcatatcctct |
| *TPS1* | Sesquiterpene cyclase | tgctggcaccatgttctctc | tcgtcccacatctcaaccaa |

|  |
| --- |
